# Broken Epidural Catheter After Vaginal Delivery

**DOI:** 10.7759/cureus.55013

**Published:** 2024-02-27

**Authors:** Abdulrahman Alfadhel, Hafiza Turkistany, Amer A Alkinani, Nasib Kabbani, Alwateen F Alabdullah

**Affiliations:** 1 Obstetrics and Gynecology, King Abdulaziz Medical City, Riyadh, SAU; 2 Anesthesia, King Abdulaziz Medical City, Riyadh, SAU

**Keywords:** broken epidural catheter, epidural catheter, vaginal delivery, delivery, epidural, catheter

## Abstract

The breakage of the epidural catheter is an alarming, rare, yet well-known complication. Despite the advances in modern imaging technologies, visualization of the broken catheter remains challenging, and surgical intervention might be necessary to remove the broken catheter. We report a case of a broken epidural catheter post-vaginal delivery, which was managed by surgical intervention.

## Introduction

The placement of an epidural catheter is a common practice in obstetric patients for pain management. Catheter insertion is associated with multiple complications such as abscess, hematoma, migration, kinking, and radiculopathy. Removing the catheter is mostly a smooth procedure but can rarely be complicated by the breakage of the catheter, with an incidence rate of 0.002% [1]. The type of catheter, the level of experience, the position of the patient during removal, the way of removing the catheter, and the patient's BMI are considered the most causative factors [2]. Due to the lack of data in the literature, management remains controversial in deciding between surgical and conservative management. The consensus nowadays is that surgical management is indicated if the patient is symptomatic. Otherwise, conservative management is recommended [3].

## Case presentation

A 23-year-old gravida 2 para 1 female with a weight of 117 kg, BMI of 46, ASA status of 1, and gestational age of 40 weeks + 2 days was admitted in labor for vaginal delivery. The patient requested an epidural for the labor pain. Under the aseptic technique, the Arrow® epidural catheter was inserted in a sitting position at the level of the L3-L4 using an 18-gauge needle. The depth was 9.5 cm with a negative aspiration test and successful from the first attempt. Taking into consideration the patient's weight and BMI, the removal of the epidural catheter was deferred to anesthesiology. The patient progressed and delivered vaginally with adequate pain control, and her delivery was unremarkable.

Post-delivery, the patient was seen by the anesthesiologist. A trial of removing the epidural catheter was started. He started to flush the catheter with normal saline without any resistance and then slow traction on the catheter was applied. He was able to pull 3 cm of the catheter and then, suddenly, the catheter tore inside the patient.

The obstetric, anesthesia, and neurosurgery teams were involved in this patient's care. The anesthesiologist disclosed the complication to the patient, and she received complete counseling, including all the management options, and all risks were explained. She underwent a pelvic X-ray, CT, and MRI scan to locate the missing epidural catheter.

**Figure 1:**
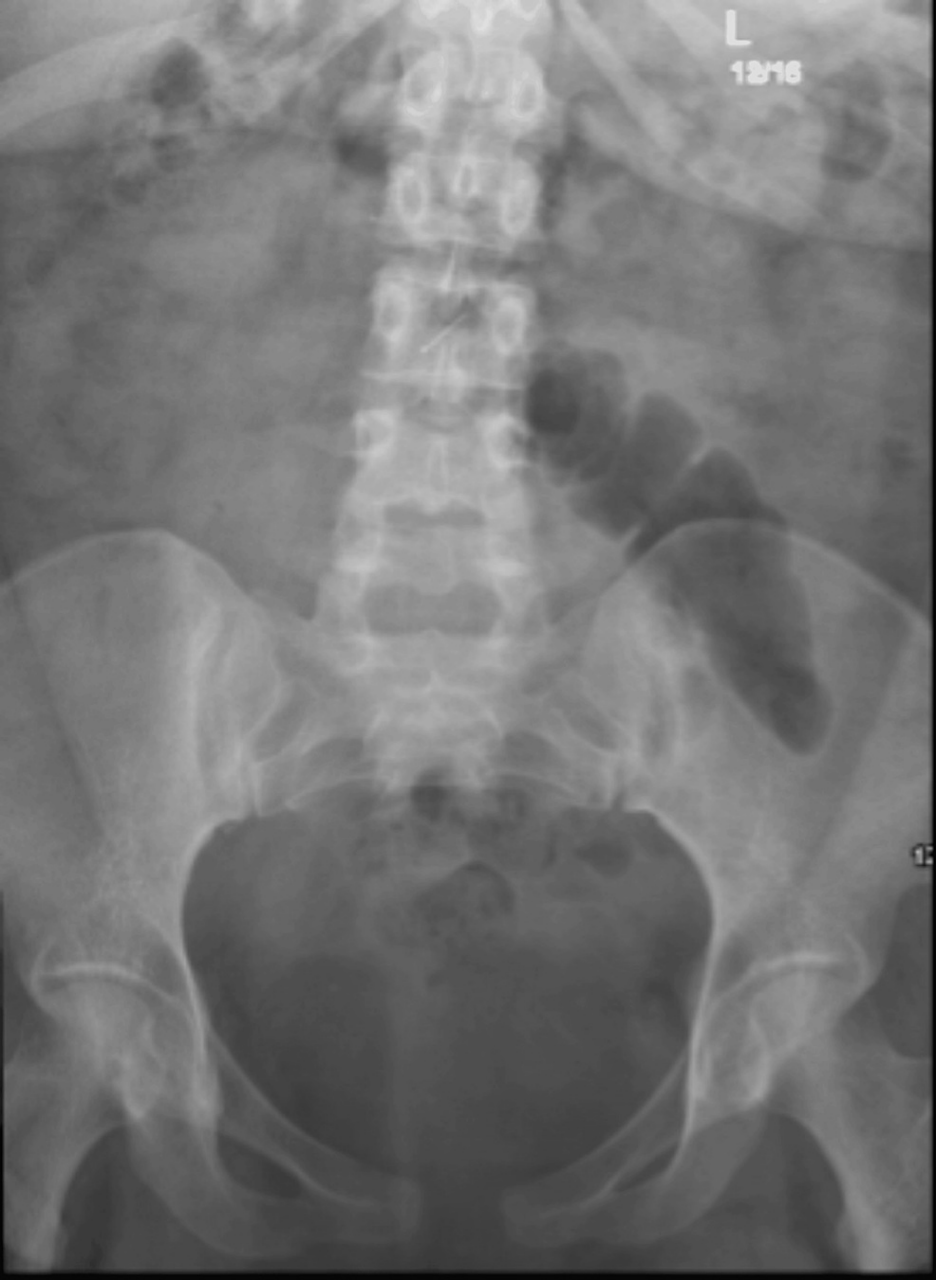
X-ray of the spine. The missing epidural catheter was not detected by X-ray.

 The X-ray findings revealed that there was mild straightening of the lumbar spine, the alignment and vertebral body height were maintained, and no fractures were noted (Figure 1).

**Figure 2:**
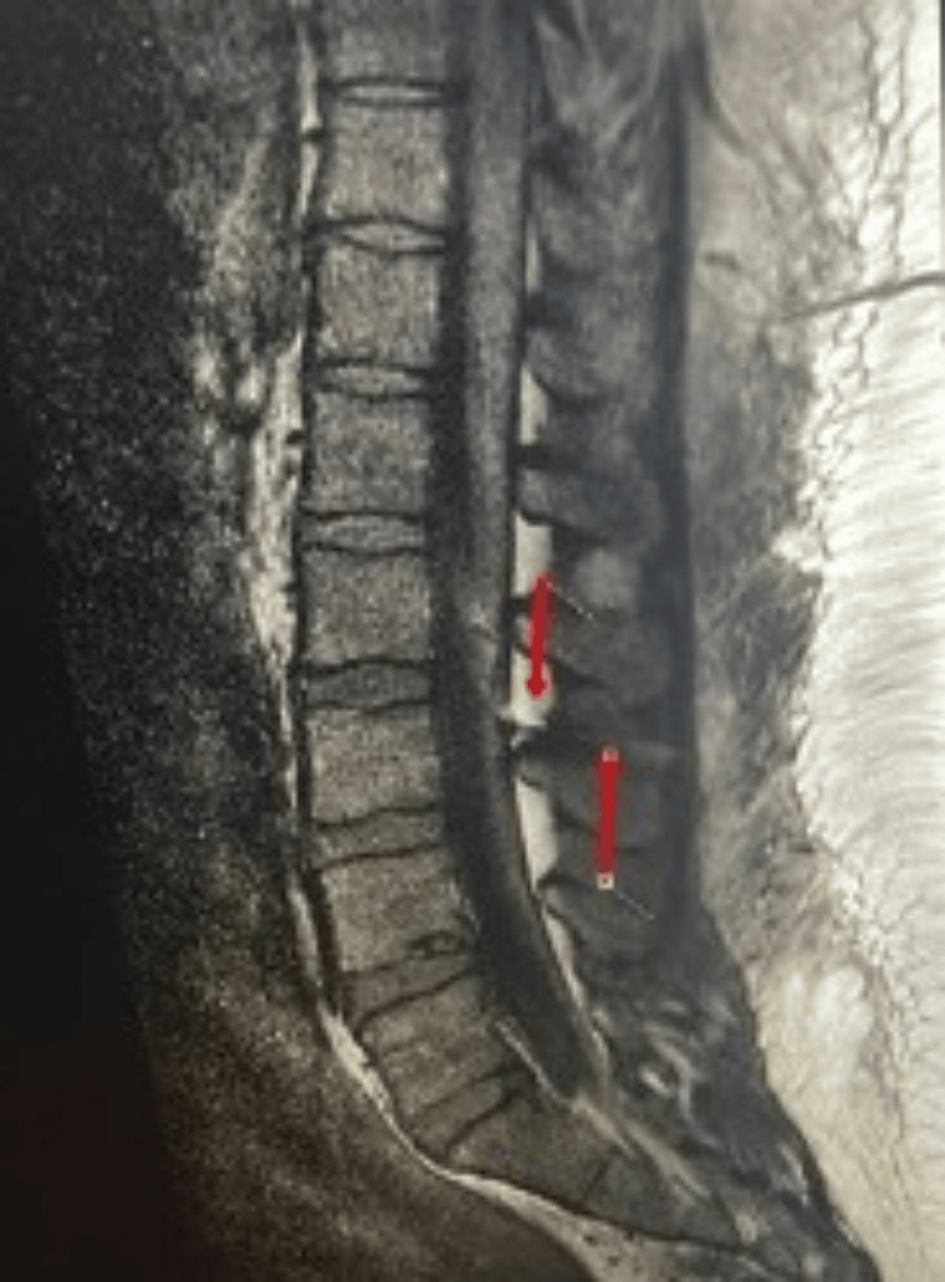
MRI of the spine. The epidural catheter is seen at the L2-L3 level, with its looped intrathecal component. The tip likely ends at the left L2-L3 foramina.

The patient’s MRI (Figure 2) showed the following: the epidural catheter is seen at the L2-L3 level, with its looped intrathecal component, the tip likely ends at the left L2-L3 foramina, the vertebral body height and alignment are maintained, the conus is intact, the marrow signal is maintained, the intervertebral discs are maintained, and the paravertebral gross tissue appears unremarkable.

**Figure 3:**
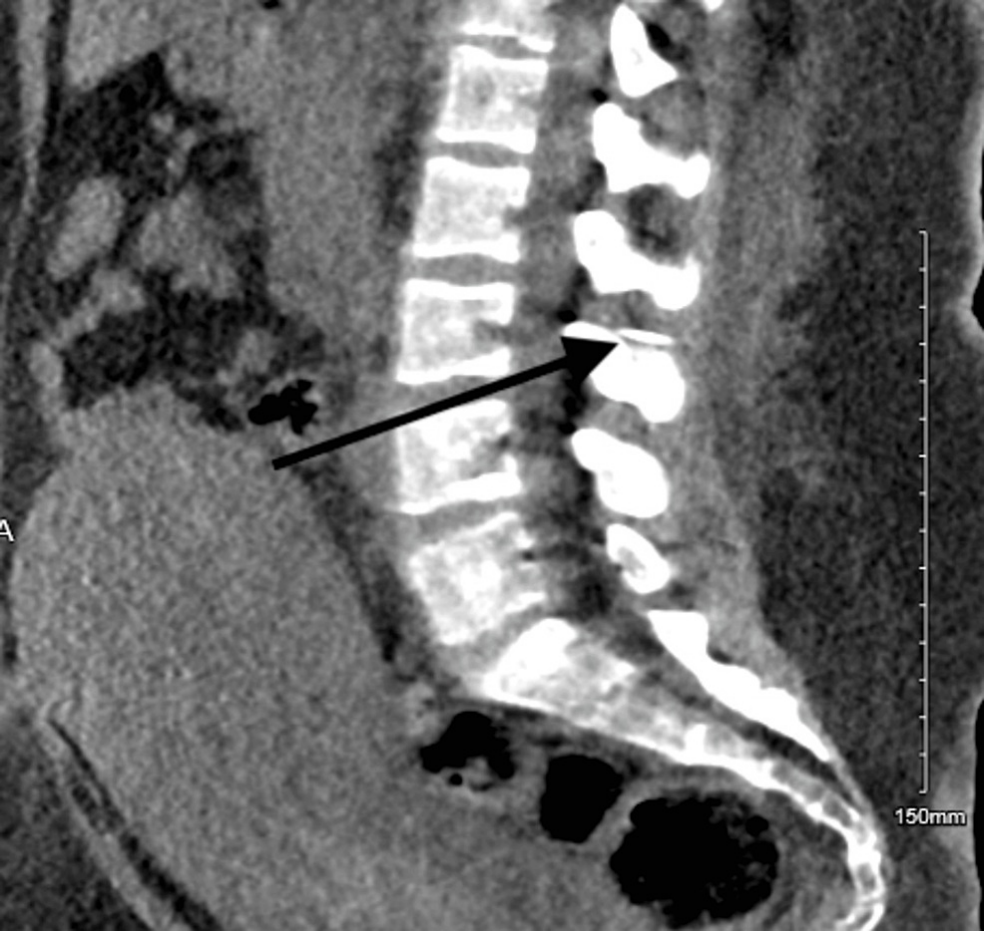
CT scan of the spine at the lumbar (at the L4 level). The CT imaging sequence indicates the epidural catheter's entrance and where it ends. In this image, you can see the catheter ending at the level of the L4 endplate.

**Figure 4:**
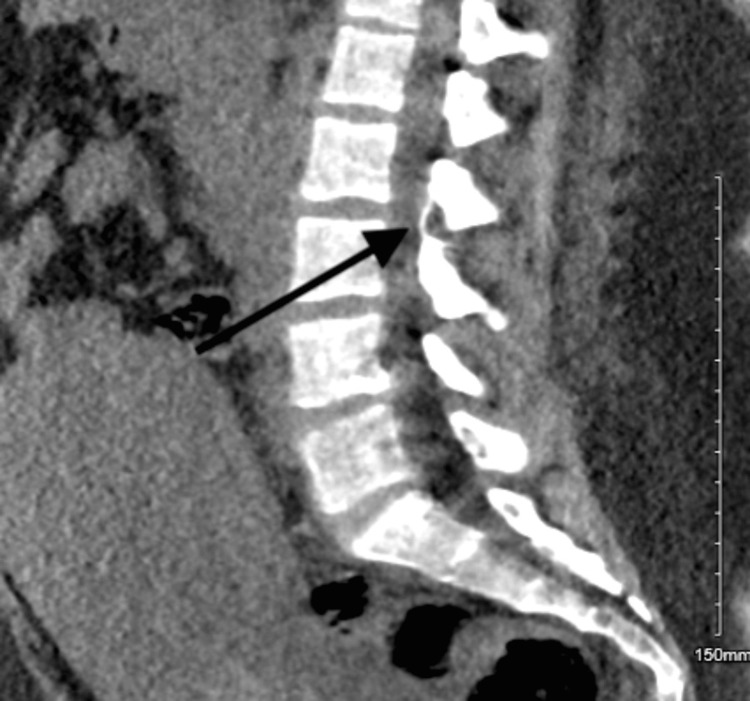
CT scan of the spine at the lumbar (spinal canal). The sequence of the CT imaging indicates the entry and the end of the epidural catheter. It can be noted it enters the spinal canal and goes upward within the posterior aspect of the spinal canal, spanning 1.7 cm.

**Figure 5:**
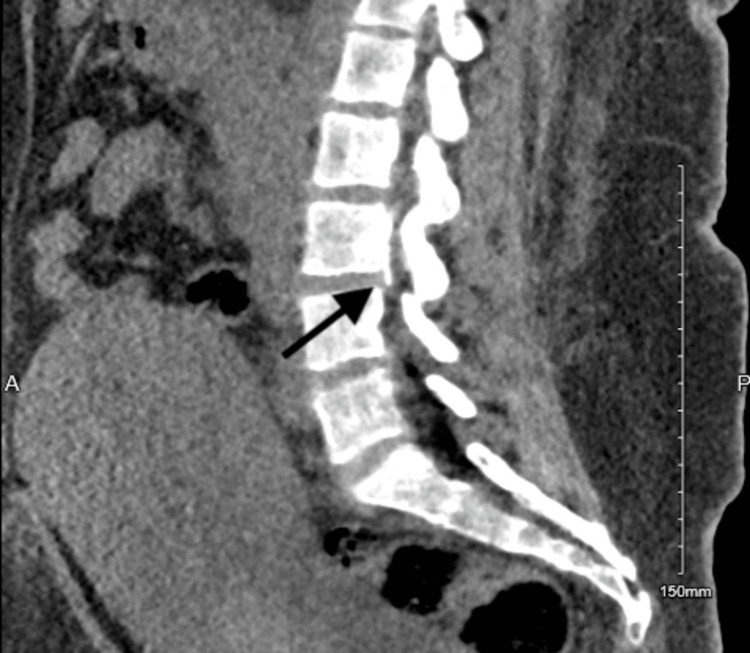
CT scan of the spine of the lumbar (at the L2 level). The CT scan sequence indicates the epidural catheter's entry and where it ends. Here you can note that one is superficial at the level of the L2 vertebra, measuring 1.2 cm, and the other is deep into the paraspinous muscle with extension to the spinal canal at the L2-L3 level.

The CT scan of the lumbar (Figures 3-5) revealed the following. There are two radiopaque linear structures. One is superficial at the level of the L2 vertebra, measuring 1.2 cm, and the other is deep into the paraspinous muscle with extension to the spinal canal at the level of L2-L3. Once it enters the spinal canal, it goes upward within the posterior aspect of the spinal canal, spanning 1.7 cm. It courses downward along the left anterolateral aspect of the spinal canal for 4.5 cm, ending at the level of the L4 endplate.

**Figure 6:**
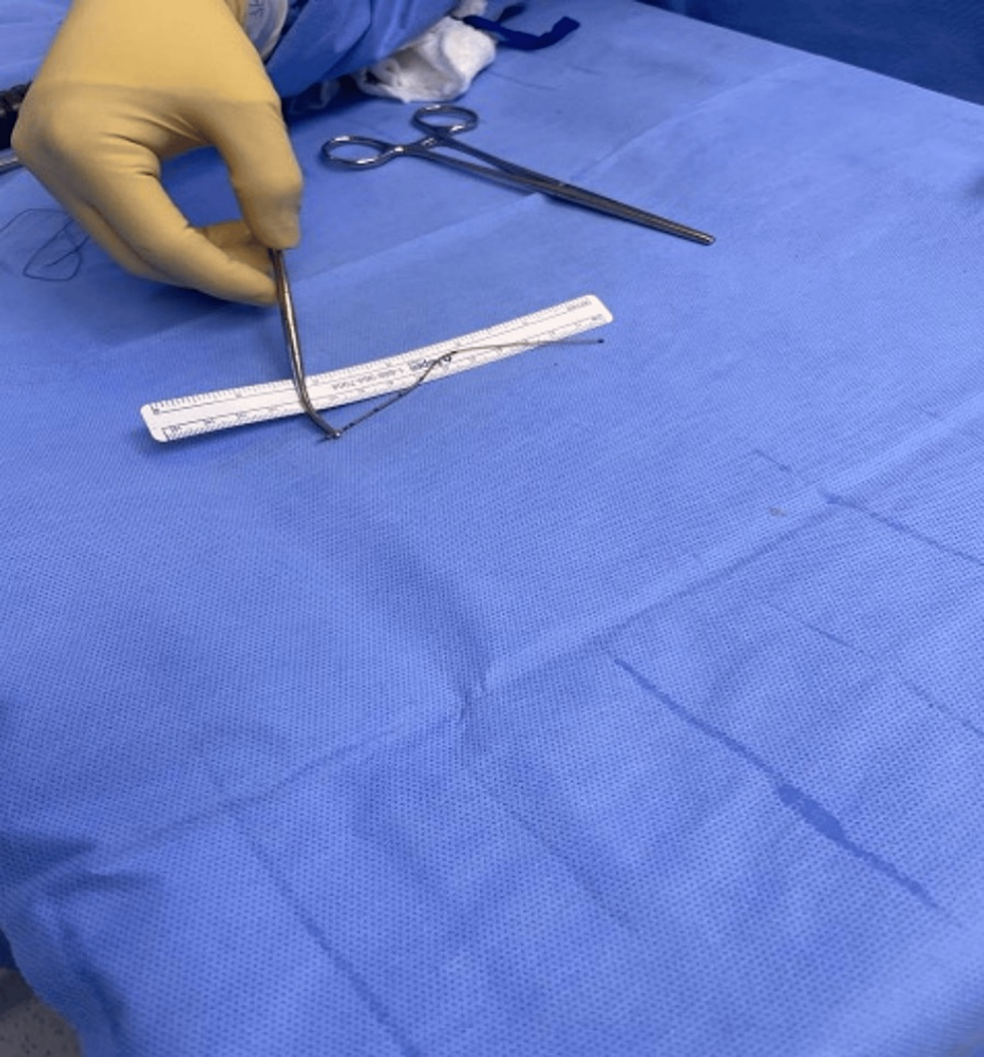
The epidural catheter. You can note the epidural catheter after successful removal.

The patient was counseled regarding the management options. It was explained to the patient that the management surgically or medically in such a case remains controversial. Surgical management is kept for symptomatic patients, while medical management in the form of follow-up is preferred in her case, as the catheter material is considered physiologically inert, no immunological reaction is expected, and fibrosis will form around it. This will not eliminate her chances of receiving a future epidural catheter. Surgical management is preferred in symptomatic patients who suffer from motor, sensory, or both symptoms in the form of laminectomy. The risk of thrombosis, infection, dural tear, spinal fluid leak, nerve injuries, and general anesthesia complications were explained to the patient. Upon her discussion with her family members, she requested surgical intervention. She underwent an exploratory laminectomy. The catheter was removed easily (Figure 6). A repeated X-ray and CT scan confirmed the complete removal of the catheter. The patient tolerated the procedure with minimal blood loss and was transferred in stable condition to the post-anesthesia care unit for observation.

The patient was observed following her surgery for 72 hours; her observation period was unremarkable, and she could resume her daily activity with no limitations and was cleared for discharge. She was granted follow-up appointments with the obstetric, anesthesia, and neurosurgery teams. All her follow-up appointments were unremarkable. She was asymptomatic and resumed her daily life activity with no limitation. She was discharged from the obstetric and anesthesia clinics and granted annual follow-up in the neurosurgery clinic.

## Discussion

The breakage of the epidural catheter is an alarming, rare, and well-known complication. This complication needs to be studied well, but the rare occurrence remains an obstacle; only isolated cases are reported. The difficulty in managing this complication is choosing the management method, as surgical or conservative approaches remain controversial.

Multiple factors were associated with this complication, such as the type of catheter, the level of experience, the position of the patient during removal, the way of removing the catheter, and the patient's BMI. In one study, a comparison between the Arrow type of catheter and the other non-reinforced catheter concluded that the Arrow type is more susceptible to stretching and breaking [4]. It is recommended whenever there is an anticipated difficulty in removing the catheter, and the catheter needs to be removed by a senior anesthesiologist. During the removal of the catheter, a slow force should be applied whenever there is resistance or stretching; this applied force should be discontinued and resumed after several hours. The patient should be placed in a similar position to the insertion position. The removal in the extreme flexion position is not effective. A higher BMI is considered one of the risk factors for the stretching and breakage of the catheter [5,6].

As this complication is poorly studied, the dilemma remains in how to manage this complication. For the management, the consensus is conservative management if the patient is asymptomatic with regular annual follow-up, as the catheter material is considered physiologically inert, and no immunological reaction is expected. Surgical management in the form of exploratory laminectomy is kept for symptomatic patients with motor or sensory function limitations [5-7].

If the conservative approach is chosen, necrotic tissue will form around the catheter. Since the catheter material is physiologically inert, we do not expect any immunological reaction. Choosing the conservative approach will not eliminate the chances of having another epidural insertion in future pregnancies, as the area can be avoided by inserting in a higher or lower level.

## Conclusions

The breakage of the epidural catheter is an alarming, rare, but not well-studied complication. Locating the missing epidural catheter remains challenging despite the advances in modern imaging technologies. The management remains controversial; the consensus is that surgical intervention is preserved for symptomatic patients, while conservative management with regular annual follow-up is recommended in asymptomatic patients.
